# Transcriptional Regulator YqeI, Locating at ETT2 Locus, Affects the Pathogenicity of Avian Pathogenic *Escherichia coli*

**DOI:** 10.3390/ani10091658

**Published:** 2020-09-16

**Authors:** Mei Xue, Yating Xiao, Dandan Fu, Muhammad Akmal Raheem, Ying Shao, Xiangjun Song, Jian Tu, Ting Xue, Kezong Qi

**Affiliations:** Anhui Province Key Laboratory of Veterinary Pathobiology and Disease Control, Anhui Agricultural University, Hefei 230036, Anhui, China; withxm@ahau.edu.cn (M.X.); xiaoyating2020@126.com (Y.X.); fudandan2020@126.com (D.F.); dr.akmalraheem1362@yahoo.com (M.A.R.); julieshao1005@163.com (Y.S.); 13275769789@126.com (X.S.); tujian1980@126.com (J.T.)

**Keywords:** avian pathogenic *Escherichia coli*, transcription regulator, YqeI, pathogenesis

## Abstract

**Simple Summary:**

Avian pathogenic *Escherichia coli* (APEC) is the causative agent of colibacillosis, threatening the development of the poultry industry. The study on APEC’s pathogenic mechanism is of great importance. In this study, we investigated the role of YqeI, a transcriptional regulator locating at *E. coli* type three secretion system 2 in APEC. The transcriptional results revealed that YqeI affected the expression of the genes involving in bacterial localization, locomotion and biological adhesion. A series experiments also demonstrated that the absence of *yqeI* decreased the bacterial flagella formation ability, motility ability, antiserum bactericidal ability, adhesion ability and colonization ability. Our data suggested that the transcriptional regulator YqeI indeed participates in the pathogenicity of APEC.

**Abstract:**

Avian pathogenic *Escherichia coli* (APEC) is the leading cause of systemic infections in poultry worldwide and has a hidden threat to public health. *Escherichia coli* type three secretion system 2 (ETT2), similar to the *Salmonella* pathogenicity island SPI1, is widely distributed in APEC and associated with virulence. The function of YqeI, which is one of the hypothetical transcriptional regulators locating at the ETT2 locus of APEC, is unknown. In this study, we successfully obtained the mutant strain AE81Δ*yqeI* of the wild type strain AE81 and performed the transcriptional profiling assays. Additionally, the transcriptional sequencing results revealed that YqeI influenced localization, locomotion and biological adhesion and so on. The transmission electron microscope observation showed that the wild type strain AE81 possessed long curved flagella, whereas the mutant strain AE81Δ*yqeI* hardly had any. The strain AE81Δ*yqeI* exhibited lower motility than AE81 after culturing the dilute bacterial suspension on a semisolid medium. It was also found that the survival ability of AE81Δ*yqeI* weakened significantly when AE81Δ*yqeI* was cultured with 0%, 10%, 20%, 30%, 40% and 50% SPF serum in PBS, and AE81Δ*yqeI* had decreased adherence to DF-1 cells compared with AE81 in the bacterial adhesion assay. The bacterial colonization assay indicated that the virulence of AE81Δ*yqeI* was reduced in the heart, liver, spleen, and lung. These results confirmed that the transcription regulator YqeI is involved in APEC’s pathogenicity, and this study provides clues for future research.

## 1. Introduction

Colibacillosis is a widespread infectious disease throughout the world [1,2,3]. It can cause systemic and local infections, and result in high mortality in poultry [4]. Avian pathogenic *Escherichia coli* (APEC) is the main cause of colibacillosis and is an important subset of extra-intestinal pathogenic *Escherichia coli* (ExPEC), including uropathogenic *Escherichia coli* (UPEC) and newborn meningitis-causing *Escherichia coli* (NMEC) [5,6,7]. Some researches demonstrate that the genomes of several APEC and human UPEC strains are similar, suggesting that APEC is a potential zoonotic pathogen, which needs more public awareness [8].

The APEC possess various virulence factors, including flagella, fimbria adhesions, iron uptake systems, autotransporter genes and a K1 capsule [9]. Two-component systems, quorum sensing systems and secretion systems also participate in regulating or exporting virulence factors in the pathogenic process of APEC [10,11,12]. The *E. coli* Type III secretion system 2 (ETT2) is one of three secretion systems (T3SSs) and resembles *Salmonella* pathogenicity island SPI1 [13]. It was first discovered by analyzing the genome sequence of enterohemorrhagic *E. coli* (EHEC) and widely prevalent in *E. coli* isolated from humans and animals, such as UPEC and APEC [14,15]. The intact ETT2 cluster encodes at least thirty-five open reading frames, including *yqe* (ecs3703-3706), *yge* (ecs3707-3712), *epr* (ecs3716-3719), *etr* (ecs3720), epa (ecs3721-3726) and eiv (ecs3727-3734) [16].

Among the thirty-five open reading frames, there are five hypothetical transcriptional regulators, including YqeI, YgeH, YgeK, EtrA and EivF [16]. It has been reported that the five hypothetical transcriptional regulators might form a regulatory circuit for bacterial colonization in EHEC [17]. The transcription regulator YqeI is closely related to MarT belonging to the *toxR*-like regulatory factor family, where *marT* is a transcriptional activator in the control of the expression of an extracellular matrix adhesin gene *misL* in *Salmonella* [18]. However, whether the transcriptional regulator YqeI affects the virulence of APEC has not been studied. In this study, we constructed the mutant strain AE81Δ*yqeI* and performed a transcriptional profiling assay, motility assay, serum bactericidal assay, bacterial adhesion assay and animal infection experiments in vivo to explore the pathogenic mechanism and regulatory pathway of transcriptional regulator YqeI in APEC.

## 2. Materials and Methods

### 2.1. Bacterial Strains, Plasmids and Growth Conditions

The clinical strain AE81 was isolated from the lung of a dead chicken with septicemic symptoms of colibacillosis in Anhui, China. The genomic DNA was extracted from AE81 using a High Pure PCR Template Preparation Kit (Roche Diagnostic, Indianapolis, IN, USA). Whole-genome sequencing was performed on the Illumina MiSeq. Hybrid de novo assembly was performed using Unicycler (v0.4.8), and the genome was annotated using the RAST server [19]. The sequence type was determined through the MLST web server [20].

### 2.2. Antimicrobial Susceptibility Testing of AE81

The minimal inhibitory concentrations (MICs) of cefuroxime, cefuroxime axetil, piperacillin and tazobactam, cefotetan, piperacillin, cefazolin, ceftazidime, ceftriaxone, cefepime, aztreonam, imipenem, meropenem, amikacin, gentamicin, tobramycin, ciprofloxacin, levofloxacin, nitrofurantoin and sulfamethoxazole/trimethoprim were determined by a Vitek 2 compact system (BioMérieux, Marcy-l’Étoile, France) with an AST-GN09 card (bioMérieux) following the manufacturer’s instructions. The results were estimated following the Clinical and Laboratory Standards Institute (CLSI) guidelines. *Escherichia coli* J53 was used as a quality control.

### 2.3. Construction of the Mutant Strain and the Complemented Strain

As previously described, the *yqeI* gene was knocked out using the lambda Red homologous recombinant system [21]. We used pKD3 as the template to amplify the chloramphenicol resistant target segments with the pKD3-*yqeI* primers and used AE81 as the template to amplify the upstream and downstream of *yqeI* with the *yqeI*-up-cm primers and *yqeI*-down-cm primers, respectively. The upstream and the downstream target fragment and the chloramphenicol *yqeI*-up-cat-down were used as the template to amplify the target fragment *yqeI*-up-cat-down. The amplified PCR product was extracted with the SanPrep Column DNA Gel Extraction Kit (Sangon Biotech, Shanghai, China). The chloramphenicol resistant fragment was transformed into the AE81 cells with pKD46 plasmids by electroporation (200 Ω, 2500 V) using the Gene Pulser Xcell (Bio Rad, Hercules, CA, USA). The mutant strains were selected using the lysogeny broth solid medium supplemented with chloramphenicol at 30 µg/mL and identified using polymerase chain reaction (PCR). The identification of the mutant strain AE81Δ*yqeI* was performed with the *yqeI*-out primers following PCR parameters: 94 °C for 4 min; 30 cycles of 94 °C for 30 s, 56 °C for 30 s and 72 °C for 1 min 30 s; 72 °C for 10 min. The temperature-sensitive plasmid pCP20 was used to eliminate chloramphenicol resistant gene fragments. Similarly, the complement strain was constructed by using recombination pSTV28 plasmids. The strains and plasmids are listed in Table 1, and the primers are listed in Table 2.

### 2.4. Detection of Growth Curve

The growth curves of AE81, AE81Δ*yqeI* and AE81Δ*yqeI*-pCm*yqeI* were determined in LB medium at 37 °C and 150 RPM. The bacteria were cultured overnight, and then they were transferred into fresh LB broth at a ratio of 1:100, respectively. Optimal density at 600 nm was monitored every hour by a spectrophotometer.

### 2.5. RNA Extraction and Transcriptional Profiling Assay

The bacterial total RNA of AE81 and AE81Δ*yqeI* was extracted when the optical density at 600 nm (OD_600nm_) were about 1.0 with a RNeasy Mini Kit (Cat#74106, Qiagen, Hilden, Germany). The RNA was purified with an RNA Clean XP Kit (Cat A63987, Beckman Coulter, Inc., Brea, CA, United States) and a RNase-Free DNase Set (Cat#79254, Qiagen, GmbH, Berlin, Germany) and examined with a Nanodrop ND-2000 spectrophotometer and Agilent Bioanalyzer 2100 (Agilent Technologies, Santa Clara, CA, USA). Then, the sequencing libraries were generated, and the fragments were purified with the AMPure XP system (Beckman Coulter, Beverly, Massachusetts, United States). The Agilent Bioanalyzer 2100 system (Agilent, Palo Alto, California, United States) was used to assess the library quality. Tag-coded samples were clustered on a cBot Cluster Generation System; then, the library preparations were sequenced, and the qualified clean data were used for bioinformatics analysis. The complete genome sequence of *E. coli* O157:H7 (NC_002695.2) was downloaded from the National Center for Biotechnology Information (NCBI, Bethesda, MD, USA) database and used as a reference genome.

### 2.6. Differential Expression Analysis

The sequence library preparation and construction were sequenced using the Illumina Hiseq sequencing platform. The number of reads mapped to each gene was counted using HTSeq. The resulting *p*-values were adjusted using the Benjamini and Hochberg approach, and *p* < 0.05 was set as the threshold for a significant difference. Gene Ontology (GO) enrichment analysis of differentially expressed genes (DEGs) was implemented using the GO seq R package. The Kyoto encyclopedia of genes and genomes (KEGG) pathway was analyzed by mapping in KGEE mapper. The data of transcriptome sequencing was deposited into the NCBI Gene Expression database with the SRA accession number: SRP238884.

### 2.7. Detection of Transcription Level by Quantitative Real-Time PCR

The transcription level of some flagella associated genes (*flhA*, *flhB*, *fliN*, *flgE*, *cheY*, *cheW* and *motB*) was detected using quantitative real-time PCR (qRT-PCR). The total volume of the reaction system was 20 µL with one µg total RNA, and the reverse transcription was conducted using SYBR green PCR master mix (Applied Biosystems, Foster City, CA, USA). The standard cycling parameters were performed on the ABI StepOne Plus instrument, and each target gene was examined three times. The primers of the target genes (*flhA*, *flhB*, *fliN*, *flgE*, *cheY*, *cheW* and *motB*) and the reference gene *dnaE* were listed in Table 2.

### 2.8. Micromorphology Observation by Transmission Electron Microscopy

The micromorphology of AE81, AE81Δ*yqeI* and AE81Δ*yqeI*-pCm*yqeI* was observed using transmission electron microscopy. After being stationary cultured for 12 h at 37 °C in LB and washed with phosphate buffer saline (PBS) three times, the bacteria were loaded onto a 200 mesh Formvar-coated copper microscopy grid (Electron Microscopy China, Beijing, China) and briefly incubated at room temperature. Then, the bacteria were stained with 2% aqueous uranyl acetate for 30 s and dried. The electron microscope (HitachiHT-7700, Hitachi, Tokyo, Japan) was used to observe the bacterial micromorphology.

### 2.9. Evaluation of Motility on Semisolid Medium

The overnight culture of AE81, AE81Δ*yqeI* and AE81Δ*yqeI*-pCm*yqeI* was each diluted 1:25 and grown to OD_600nm_ at 0.4. Each culture was washed with PBS and diluted 1:100, then plated on the LB plates with 0.3% agar [22]. The plates coating with the dilution were cultured for eight hours at 37 °C, then the motile cycles of the colonies were observed.

### 2.10. Serum Bactericidal Assay

The overnight cultures of AE81, AE81Δ*yqeI* and AE81Δ*yqeI*-pCm*yqeI* were diluted 1:25 with LB broth medium and grown until they reached an OD_600_ of 0.6. Specific pathogen free (SPF) chicken sera were diluted to 0%, 10%, 20%, 30%, 40% and 50% in phosphate buffer saline (PBS). Then AE81, AE81Δ*yqeI* and AE81Δ*yqeI*-pCm*yqeI* were incubated with 0%, 10%, 20%, 30%, 40% and 50% diluted sera, respectively. After culturing for 2 h at 37 °C, the growth rate for each plate was determined using a multimode microplate reader, and OD_600nm_ was measured every hour.

### 2.11. Bacterial Adhesion Assay

When the bacterial OD_600nm_ was at 1.0, AE81, AE81Δ*yqeI* and AE81Δ*yqeI-pCm*Δ*yqeI* were centrifuged, respectively. Then, the pellet was washed three times with Dulbecco’s modified Eagle’s medium (DMEM). The washed pellet at a MOI of 100 was used to infect the chicken embryo fibroblast DF-1 cell. After coculturing the bacteria and DF-1 cell for 2 h at 37 °C with 5% CO_2_, the medium was removed and PBS was added to remove nonadherent bacteria. Finally, 0.5% Triton X-100 was added to lyse cells, and survival AE81, AE81Δ*yqeI* and AE81Δ*yqeI-pCm*Δ*yqeI* were counted by plating on LB agar plates.

### 2.12. Animal Infection Experiments In Vivo

Animal infection experiments in vivo were performed to evaluate the colonization ability of AE81, AE81Δ*yqeI* and AE81Δ*yqeI-pCm*Δ*yqeI*. Fifty 1-day-old chickens were obtained from a poultry company (Anhui Anqin, Hefei, China). The chickens can freely eat food and drink water. The Institutional Animal Care and Use Committee (IACUC) guidelines by Anhui Agricultural University (Number: 2020-012) were followed to take care of these chickens. After a week of feeding, three chickens of each group were infected using an intramuscular injection of 0.5 mL bacterial suspension with 1 × 10^8^ CFU. At 24 h postinfection, the chickens were euthanized, and samples were aseptically collected. Tissue samples of about 0.2 g—taken from the heart, liver, spleen and lung of the chicks—were weighed, triturated with sterile PBS and homogenized. Then, 10-fold serial dilutions of the homogenates were prepared and plated onto LB agar plates for culture at 37 °C.

### 2.13. Statistical Analysis

The SPSS (v19.0) software was applied to analyze the data. Between the group of AE81 and AE81Δ*yqeI*, or the group of AE81Δ*yqeI* and AE81Δ*yqeI-pCmyqeI*, the paired *t*-test was used for statistical comparisons, and the *p*-value of ≤0.05 was set as the significant statistical level.

## 3. Results

### 3.1. Genome Sequencing Analysis and Antimicrobial Susceptibility of AE81

AE81 was subjected to sequencing on the MiSeq platforms. The 4.848-Mb chromosome was obtained through a de novo hybrid assembly. Strain AE81 belonged to sequence type ST1010 and serotype O166:H45. AE81 was sensitive to all tested antibiotics, including ampicillin (4 ug/mL), ampicillin/sulbactam (≤2 ug/mL), cefotetan (≤4 ug/mL), cefepime (≤1 ug/mL), ceftazidime (≤1 ug/mL), ceftriaxone (≤1 ug/mL), imipenem (≤1 ug/mL), aztreonam (≤1 ug/mL), piperacillin and tazobactam (≤4 ug/mL), ciprofloxacin (≤0.25 ug/mL), levofloxacin (≤0.25 ug/mL), sulfamethoxazole/trimethoprim (≤20 ug/mL), nitrofurantoin (≤16 ug/mL), amikacin (≤2 ug/mL), gentamicin (≤1 ug/mL) and tobramycin (≤1 ug/mL).

### 3.2. Absence of yqeI Did Not Affect the Growth of AE81

**Figure 1:**
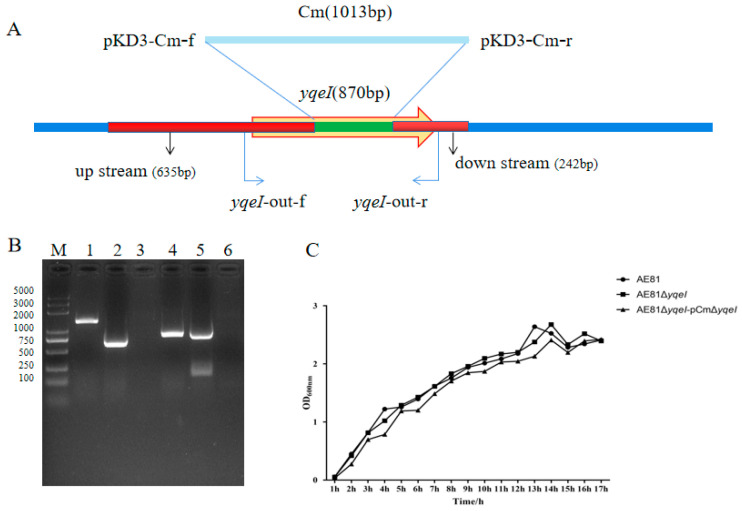
(**A**) The schematic diagram of the strategy for deleting the *yqeI* gene in AE81: (**B**) confirmation of the mutant strain AE81Δ*yqeI* and the complemented strain AE81Δ*yqeI*-pCmΔ*yqeI.* M: 5000 DNA marker; Lane 1: PCR product (1309 bp) amplified from AE81 with primers *yqeI*-out; Lane 2: PCR product (577 bp) amplified from AE81Δ*yqeI* with primers *yqeI*-out; Lane 3: negative control with primers *yqeI*-out; Lane 4: PCR product (870 bp) amplified from AE81 with primers C-*yqeI*; Lane 5: PCR product (870 bp) amplified from AE81Δ*yqeI*-pCmΔ*yqeI* with primers C-*yqeI*; Lane 6: negative control with primers C-*yqeI*; (**C**) the growth curves of AE81, AE81Δ*yqeI* and AE81Δ*yqeI*-pCmΔ*yqeI* in lysogeny broth.

The schematic diagram of the strategy for deleting the *yqeI* gene in AE81 is shown in Figure 1A. The mutant strain AE81Δ*yqeI* and the complemented strain AE81Δ*yqeI-pCm*Δ*yqeI* were successfully constructed (Figure 1B), and their growth curve was like that of the wild type strain AE81 (Figure 1C).

### 3.3. YqeI Participated in Multiple Bacterial Biological Pathways

The transcriptomic sequencing results showed that YqeI affected 587 differentially expressed genes (DEGs) (Table S1). The differentially expressed genes, including 391 up-regulated and 196 down-regulated genes, were mainly involved in biological processes, cellular components and gene molecular function. It was noteworthy that the biological process covered localization, locomotion and biological adhesion (Figure S1). In addition, the most enriched KEGG classification was global and overview maps. Other major enrichments included cellular processes and environmental information process. In addition, the cell motility pathway was one of the most enriched KEGG pathways (Figure S2).

### 3.4. YqeI Up-Regulated the Expression of Flagella-Related Genes

**Figure 2:**
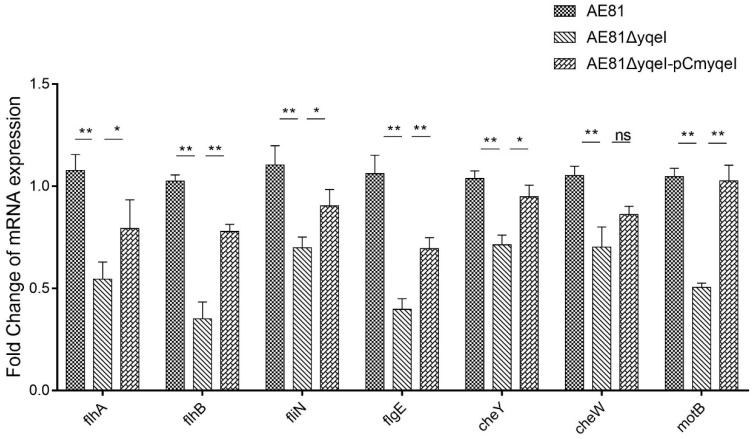
The relative expression of some flagella-related genes detected by qRT-PCR, * *p* < 0.05, ** *p* < 0.01, ns (no significant difference).

Inactivation of *yqeI* affected the expression of twenty-nine flagella-related genes (Table 3). Among the DEGs, three genes—*ybeL*, *dppA* and *malE*—were up regulated, and twenty-six genes were down regulated, including filament cap (*fliD*), filament (*fliC*), hook filament junction (*flgK*), hook (*fliK* and *flgE*), basal body (*motA* and *motB*), C ring (*fliM* and *fliN*), type III secretion system (*flhA*, *flhB*, *fliI* and *fliO*), cytoplasmic chaperone (*flgN*, *fliJ* and *fliT*), early gene products (*flhC* and *flhD*) and late gene products (*flgM*). The real-time qPCR results showed that the mRNA levels of seven genes, including *flhA*, *flhB*, *fliN*, *flgE*, *cheY*, *cheW* and *motB*, were decreased significantly in AE81Δ*yqeI* compared with AE81 (*p* < 0.01). The mRNA levels of those genes in AE81Δ*yqeI*-pCm*yqeI* recovered some, and the difference between AE81 and AE81Δ*yqeI* was also significant (*p* < 0.01) (Figure 2).

### 3.5. Inactivation of yqeI Decreased the Formation of Flagella and Motility

**Figure 3:**
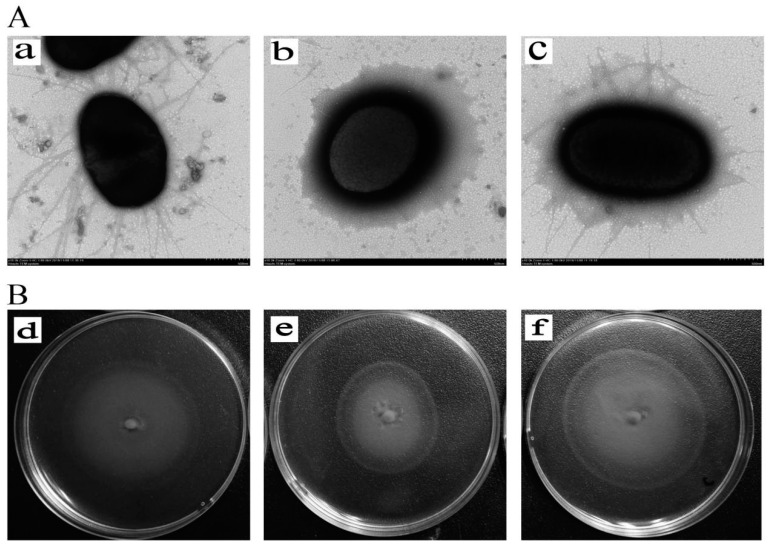
(**A**). The flagella of AE81, AE81Δ*yqeI* and AE81Δ*yqeI*-pCm*yqeI* in the transmission electron micrographs views (×10,000). (**a**) The morphological observation of AE81 (×10,000); (**b**) the morphological observation of AE81Δ*yqeI* (×10,000); (**c**) the morphological observation of AE81Δ*yqeI*-pCm*yqeI* (×10,000) (**B**). The observation of motility ability. (**d**) The motility circle of the wild strain AE81; (**e**) the motility circle of AE81Δ*yqeI*; (**f**) the motility circle of AE81Δ*yqeI*-pCm*yqeI*.

Under the view of the transmission electron microscope, the morphological structure of AE81, AE81Δ*yqeI* and AE81Δ*yqeI*-pCm*yqeI* was significantly different. Many long flagella were distributed at the periphery of AE81, but the flagella of AE81Δ*yqeI* were few and impaired. A few broken flagella appeared on the surface of AE81Δ*yqeI*-pCm*yqeI*, and the flagella formation ability was partially recovered (Figure 3A). In addition, after being cultured on a semisolid medium for eight hours, AE81, AE81Δ*yqeI* and AE81Δ*yqeI*-pCm*yqeI* all had a clear motility circle. However, the diameter halo of AE81 was significantly smaller than that of AE81Δ*yqeI*, and the diameter halo of AE81Δ*yqeI*-pCm*yqeI* was similar to that of AE81 (Figure 3B). These results indicated that the absence of *yqeI* reduced the flagella formation and motility of AE81.

### 3.6. YqeI Contributed to the Survival Ability of AE81 in the Serum Resistance

**Figure 4:**
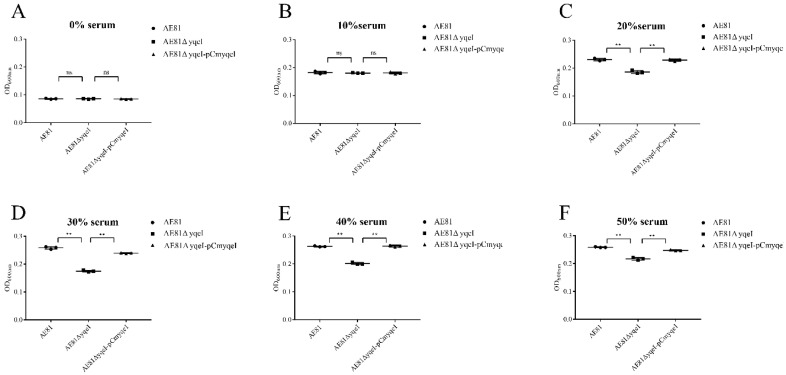
Bacterial resistance to chicken serum. Growth was determined at OD_600nm_. (**A**) SPF chicken serum was added at concentrations of 0%; (**B**) SPF chicken serum was added at concentrations of 10%; (**C**) SPF chicken serum was added at concentrations of 20%; (**D**) SPF chicken serum was added at concentrations of 30%; (**E**) SPF chicken serum was added at concentrations of 40%; (**F**) SPF chicken serum was added at concentrations of 50%.

When cultured in PBS, the survival capability of AE81, AE81Δ*yqeI* and AE81Δ*yqeI*-pCm*yqeI* was similar. However, when AE81, AE81Δ*yqeI* and AE81Δ*yqeI*-pCm*yqeI* were cultured with 10%, 20%, 30%, 40% and 50% serum for two hours, the survival capacity of AE81Δ*yqeI* significantly decreased, but the bacterial activity of AE81Δ*yqeI*-pCm*yqeI* was restored (Figure 4). The results indicated that the transcriptional regulator YqeI increased the serum resistance of AE81.

### 3.7. YqeI Affected the Adhesion Ability of AE81

**Figure 5:**
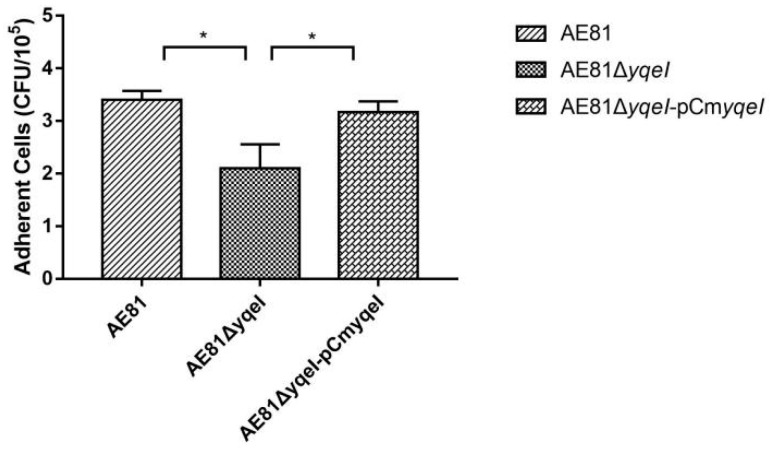
The cell adhesion number was calculated by plate counting. The cell number of AE81Δ*yqeI* was significantly decreased compared with AE81. The cell numbers of AE81 and AE81Δ*yqeI*-pCm*yqeI* were similar, * *p* < 0.05.

The bactericidal adhesion capacities of AE81, AE81Δ*yqeI* or AE81Δ*yqeI*-pCm*yqeI* were investigated by infecting the DF-1 chicken fibroblast cell line. As shown in Figure 5, the bacterial adhesion capacity of AE81Δ*yqeI* to DF-1 cells decreased compared with AE81, and the bacterial adhesion capacity of AE81Δ*yqeI*-pCm*yqeI* was restored. These results suggested that the transcriptional regulator YqeI increased the capacity of APEC to adhere to DF-1 cells.

### 3.8. Absence of yqeI Attenuated Bacterial Colonization during Systemic Infection In Vivo

**Figure 6:**
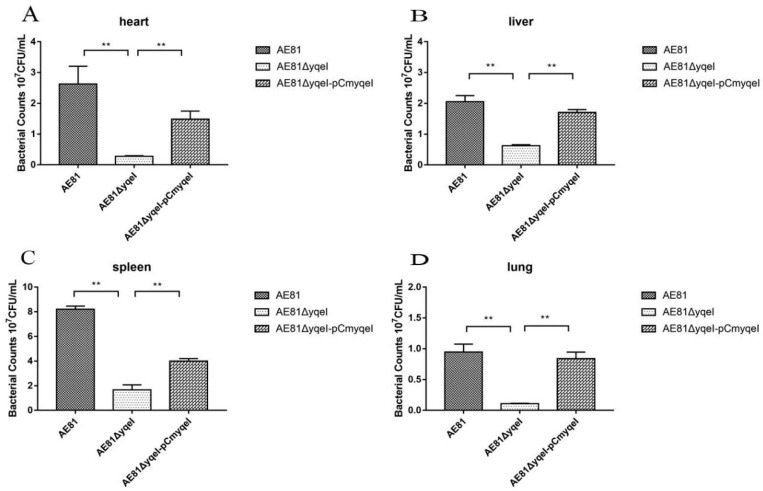
Bacterial colonization during systemic infection in chickens at 24 h post infection (**, *p* < 0.01). (**A**) The number of AE81, AE81∆*yqeI* and AE81∆*yqeI*-pCm*yqeI* was counted from the heart; (**B**) The number of AE81, AE81∆*yqeI* and AE81∆*yqeI*-pCm*yqeI* was counted from the heart liver; (**C**) The number of AE81, AE81∆*yqeI* and AE81∆*yqeI*-pCm*yqeI* was counted from the heart spleen; (**D**) The number of AE81, AE81∆*yqeI* and AE81∆*yqeI*-pCm*yqeI* was counted from the heart lung.

Chickens were intramuscularly injected with AE81, AE81Δ*yqeI* and AE81Δ*yqeI*-pCm*yqeI*, respectively, and developed clinical signs including reduced food intake, lack of energy and diarrhea. The bacterial levels in the heart, liver, spleen and lung tissues of infected chicks were determined 24 h postinoculation. The bacterial loads of AE81 in the infected heart, liver, spleen and lung were 2.63 × 10^7^ CFU, 2.05 × 10^7^ CFU, 8.20 × 10^7^ CFU and 9.47 × 10^6^ CFU per gram of tissue, respectively; the bacterial loads of AE81Δ*yqeI* were 2.77 × 10^6^ CFU, 6.23 × 10^6^ CFU, 1.67 × 10^7^ CFU and 1.10 × 10^6^ CFU, respectively; the bacterial loads of AE81Δ*yqeI*-pCm*yqeI* were 1.49 × 10^7^ CFU, 1.70 × 10^7^ CFU and 4.00 × 10^7^ CFU and 8.67 × 10^6^ CFU, respectively (Figure 6). These results showed that when chickens were infected with AE81Δ*yqeI*, a reduction in the bacterial numbers in the heart, liver, spleen and lung was observed, compared to those in chickens infected with AE81 and AE81Δ*yqeI*-pCm*yqeI*, indicating that YqeI plays a significant role in APEC virulence and may regulate virulence factors required for systemic infection in the chick infection model.

## 4. Discussion

Previous studies have confirmed that intact ETT2 or ETT2 components play a role in the pathogenicity of *E. coli*. In UPEC, an intact ETT2 cluster has a global effect on the cell surface, drug resistance, motility, serum resistance and the secretion of extracellular proteins and outer membrane vesicles [23]. In APEC, the transcriptional regulator *etrA* significantly affects bacterial survival in HD-11 macrophages, virulence levels in ducks, fimbriae development and so on [24]. In the meningitis-causing *E. coli* K1 strain EC10 (O7:K1), ETT2 and EivA are involved with host adhesion and invasion, and play important roles in pathogenesis [25]. In EHEC O157:H7, EtrA and EivF decrease adhesion to human intestinal epithelial cells [17]. In this study, it was proved that the hypothetical transcriptional regulator YqeI, which is located in the ETT2 locus, decreased the flagella formation, motility ability, serum resistance, cell adhesion ability and bacterial colonization ability, supporting that the transcriptional regulator YqeI might play an important role in the pathogenicity of APEC.

The flagellum is a motor organelle and a protein export apparatus, controlling bacterial motility and behavior [26]. Many factors have been reported to be capable of affecting the flagella assembly, such as the two-component system PhoP/Q [27], the global transcriptional regulator YjjQ [28] and the type VI secretion system component IcmF [29] and ETT2 locus. In UPEC, in the absence of ETT2, the secretion of FliC, FliD, FlgK and FlgM through the flagella T3SS was decreased [23]. In this study, when deleting *yqeI*, the flagellum of AE81 was broken, and the motility of AE81 decreased significantly, and *fliC*, fliD and flgK and flgM all had lower transcription levels. These results suggested that the transcriptional regulator YqeI might attenuate the flagella formation and ability by affecting the motility pathway. Therefore, the flagella assembly can be regulated by the two-component systems, the global transcriptional regulators and the secretion systems, and this study contributes to the understanding of the flagella regulatory mechanism.

Septicemia, one of the typical symptoms caused by APEC, has a connection with the serum resistance [30]. Type 1 fimbriae and outer membrane protein (such as OmpA, TraT and Iss) are both important factors contributing to serum resistance [31]. In APEC, several regulatory proteins which are associated with serum resistance have been reported. RfaH, a regulatory protein, might affect the serum susceptibility by decreased expression of traT and iss [32]. This study proved that the transcriptional regulator YqeI significantly increased the bactericidal antiserum ability by affecting the gene expression of the type I fimbriae. However, it was reported that another ETT2 transcriptional regulator EtrA did not affect the serum resistance. The transcriptional regulator YqeI was the first ETT2 transcriptional regulator participating in the serum resistant.

In addition, type 1 fimbriae are one of the best known adhesins present among APEC [33]. Adhesins are the specific receptor sites on the bacterial surface and mediate the bacterial attachment to the cell surface [34]. The transcription sequencing revealed that YqeI decreased the expression of type I pilus adhesin gene *fimH*, type I pilus component gene *fimG*, major type I subunit *fimF*, *fimD* and *ycbU*, proving that the down-expressed fimbriae-related genes might account for the diminished adhesion ability and colonization. This study evaluated the functional role of the transcription regulator YqeI in APEC and confirmed that the transcriptional regulator YqeI affects the virulence of APEC, and this might provide theoretical support for the development of new therapeutic strategies.

## 5. Conclusions

This study confirmed that the absence of the transcription regulator YqeI attenuated bacterial flagellum formation ability, motility ability, survival ability in serum, adhesion ability and bacterial colonization ability. It supported that the transcriptional regulator YqeI participates in the pathogenicity of APEC and provided insight for further mechanism research of APEC’s pathogenicity.

## Figures and Tables

**Table 1:** Strains and plasmid used in this study.

Strains or Plasmid	Genotype or Description	Source
Strains		
AE81	APEC clinical strain, isolated from lung	Laboratory stock
AE81Δ*yqeI*	AE81 *yqeI* deletion mutant	This study
AE81Δ*yqeI*-pCm*yqeI*	AE81Δ*yqeI* with the plasmid pCm*yqeI*, Cmr ^1^	This study
Plasmid		
pCm*yqeI*	pSTV28 with *yqeI* gene, Cmr ^1^	This study

^1^ Cmr, chloramphenicol-resistant.

**Table 2:** Primer used in qPCR.

Primer	Sequence (5′-3′)	Product Length (bp)
*yqeI-up-cm*	f: TGATGGTCTTGATGTTGCC r: CCAGCCTACAGCTCAGGCCAGAACTCGATA	635
*yqeI-down-cm*	f: TATTCATATGTAACCCTGTCTTACCGTG r: TTCCACTCAACGCAACAG	242
*pKD3-yqeI*	f: TGGCCTGAGCTGTAGGCTGGAGCTGCTT r: GACAGGGTTACATATGAATATCCTCCTTAG	1033
*C-yqeI*	f: CCGGAATTCCATGTACTGGATTATTAACGA r: CGCGGATCCTCAACCACGATTAACCTCAC	870
*yqeI-out*	f: TTCGGTCCAACATTGATA r: TAAGATTTGCTCGTCCCT	1309
*flgE-rt*	f: TCAGTCGGACGACATGGTAGAT r: CGAGTATGTTGCGAATGTGGAT	148
*fliN-rt*	f: CGGTGGTGATGTCAGCGG r: GCTCTTTGATGGTCATTCGTGTG	104
*flhA-rt*	f: GAAGTGGTGGTCGTTGCCGATAAA r: GCTGGAGTTGAAGATAATGGGGG	121
*flhB-rt*	f: CCTACCAAATCCATCGCATTACCC r: CTCCTGGTTGGCAGCGTGA	113
*cheY-rt*	f: TAACACTGGCATTGCCGACATCG r: AATGTTGAGGAAGCGGAAGATGGC	159
*motB-rt*	f: GGCGCTTACTGGCTCATTCTGG r: GCCGTCAACCGTCGCATCAG	99
*cheW-rt*	f: CACATCCACCTGGCTGAACT r: AAAGTGCAGGAGATCCGTGG	128

**Table 3:** Differentially expressed genes in the cell motility pathway.

Genes	Symbol	Description	Log2FoldChange
Ecs_0681	*ybeL*	methyl-accepting chemotaxis protein II, aspartate sensor receptor	1.50
Ecs_1448	*flgN*	flagella synthesis protein FlgN	−1.29
Ecs_1449	*flgM*	negative regulator of flagellin synthesis FlgM	−1.43
Ecs_1454	*flgE*	flagellar hook protein FlgE	−1.06
Ecs_1460	*flgK*	flagellar hook-associated protein 1 FlgK	−1.23
Ecs_2589	*flhA_2*	flagellar biosynthesis protein FlhA	−1.54
Ecs_2590	*flhB*	flagellar biosynthetic protein FlhB	−1.32
Ecs_2591	*cheZ*	chemotaxis protein CheZ	−1.69
Ecs_2592	*cheY*	two-component system, chemotaxis family, chemotaxis protein CheY	−1.67
Ecs_2596	*tsr_1*	methyl-accepting chemotaxis protein II, aspartate sensor receptor	−1.14
Ecs_2597	*cheW*	purine-binding chemotaxis protein CheW	−1.03
Ecs_2599	*motB*	chemotaxis protein MotB	−1.20
Ecs_2600	*motA*	chemotaxis protein MotA	−1.48
Ecs_2601	*flhC*	flagellar transcriptional activator FlhC	−1.39
Ecs_2602	*flhD*	flagellar transcriptional activator FlhD	−1.20
Ecs_2662	*fliC*	flagellin	−1.60
Ecs_2663	*fliD*	flagellar hook-associated protein 2	−1.04
Ecs_2665	*fliT*	flagellar protein FliT	−1.14
Ecs_2680	*fliI*	flagellum-specific ATP synthase	−1.00
Ecs_2681	*fliJ*	flagellar FliJ protein	−2.53
Ecs_2682	*fliK*	flagellar hook-length control protein FliK	−1.23
Ecs_2684	*fliM*	flagellar motor switch protein FliM	−1.47
Ecs_2685	*fliN*	flagellar motor switch protein FliN/FliY	−2.61
Ecs_2686	*fliO*	flagellar protein FliO/FliZ	−2.82
Ecs_3042	*mglB*	methyl-galactoside transport system substrate-binding protein	2.13
Ecs_4424	*dppA*	dipeptide transport system substrate-binding protein	2.18
Ecs_4437	*yiaD*	OmpA-OmpF porin, OOP family	−1.17
Ecs_5017	*malE*	maltose/maltodextrin transport system substrate-binding protein	2.82
Ecs_5315	*tsr*	methyl-accepting chemotaxis protein I, serine sensor receptor	−1.51

## Supplementary Materials

The following are available online at https://www.mdpi.com/2076-2615/10/9/1658/s1, Figure S1: GO enrichment analysis of significantly DEGs. The abscissa is the number of significant DEGs, and the ordinate indicates the GO terms. There are three main categories of GO terms, each marked with a different color. Figure S2: The KEGG pathway classification map of significantly DEG. The abscissa is the number of significant DEGs, and the ordinate indicates the pathway classification. The different pathway types are annotated with different colors. Table S1: The differently expressed genes between the wild strain AE81 and the mutant strain AE81∆*yqeI*.
